# Trends and patterns of air quality in Santa Cruz de Tenerife (Canary Islands) in the period 2011–2015

**DOI:** 10.1007/s11869-017-0484-x

**Published:** 2017-05-17

**Authors:** Jose M. Baldasano, Jordi Massagué

**Affiliations:** 1 Earth Sciences Department, Barcelona Supercomputing Center-Centro Nacional de Supercomputación, Barcelona, Spain; 2 Environmental Modeling Laboratory, Technical University of Catalonia, Barcelona, Spain

**Keywords:** Air quality, Santa Cruz de Tenerife (Canary Islands), SO_2_ pollution, NO_x_-O_3_ interactions, Trends and patterns, Mineral dust

## Abstract

Air quality trends and patterns in the coastal city of Santa Cruz de Tenerife (Canary Islands, Spain) for the period 2011–2015 were analyzed. The orographic and meteorological characteristics, the proximity to the African continent, and the influence of the Azores anticyclone in combination with the anthropogenic (oil refinery, road/maritime traffic) and natural emissions create specific dispersion conditions. SO_2_, NO_2_, PM_10_, PM_2.5_, and O_3_ pollutants were assessed. The refinery was the primary source of SO_2_; EU hourly and daily average limit values were exceeded during 2011 and alert thresholds were reached in 2011 and 2012. WHO daily mean guideline was occasionally exceeded. Annual averages in the three stations that registered the highest concentrations in 2011 and 2012 were between 9.3 and 20.4 μg/m^3^. The spatial analysis of SO_2_ concentrations with respect to prevailing winds corroborates a clear influence of the refinery to the SO_2_ levels. In 2014 and 2015, the refinery did not operate and the concentrations fell abruptly to background levels of 2.5–7.1 μg/m^3^ far below from WHO AQG. NO_2_ EU limit values, as well as WHO AQG for the period 2011–2015, were not exceeded. The progressive dieselization of the vehicle fleet caused an increment on NO_2_ annual mean concentrations (from 2011 to 2015) measured at two stations close to busy roads 25 to 31 μg/m^3^ (+21%) and 27 to 35 μg/m^3^ (+29%). NO_*x*_ daily and weekly cycles (working days and weekends) were characterized. An anti-correlation was found between NO_*x*_ and O_3_, showing that O_3_ is titrated by locally emitted NO. Higher O_3_ concentrations were reported because less NO_*x*_ emitted during the weekends showing a clear weekend effect. Saharan dust intrusions have a significant impact on PM levels. After subtracting natural sources contribution, none of the stations reached the EU maximum 35 yearly exceedances of daily means despite seldom exceedances at some stations. None of the stations exceeded the annual mean EU limit values; however, many stations exceeded the annual mean WHO AQG. Observed PM_10_ annual average concentrations in all the stations fluctuated between 10.1 and 35.3 μg/m^3^, where background concentrations were 6.5–24.4 μg/m^3^ and natural contributions: 4.2–9.1 μg/m^3^. No PM_10_ temporal trends were identified during the period except for an effect of washout due to the rain: concentrations were lower in 2013 and 2014 (the most rainy years of the period). None of the stations reached the PM_2.5_ annual mean EU 2015 limit value. However, almost all the stations registered daily mean WHO AQG exceedances. During 2015, PM_2.5_ concentrations were higher than the previous years (2015, 8.8–12.3 μg/m^3^; 2011–2014, 3.7–9.6 μg/m^3^). O_3_ complied with EU target values; stricter WHO AQG were sometimes exceeded in all the stations for the whole time period.

**Electronic supplementary material:**

The online version of this article (doi:10.1007/s11869-017-0484-x) contains supplementary material, which is available to authorized users.

## Introduction

Air pollution is one of the biggest environmental risks to health. Outdoor air pollution alone kills around 3 million people worldwide each year, mainly from chronic diseases. Only one person in ten lives in a city that complies with the WHO Air Quality Guidelines (AQG). Air pollution continues to rise at an alarming rate and affects economies and people’s quality of life (WHO 2016). The cost of air pollution as recently valued by the World Bank in terms of total welfare losses for Spain in 2013 is estimated at 49,331 million dollars, which represents 3.39% of GDP (WB 2016). Air pollution is a matter of growing concern for both public administrations and citizens.

This study focuses on the coastal city of Santa Cruz de Tenerife City on the Canary Islands (SCT), where previous studies showed that there is an association between hospitalizations due to heart failure and exposure to particles in the ambient air (Domínguez-Rodríguez et al. 2011) and SO_2_ pollution (Milford et al. 2008, and Baldasano et al. 2014).

González and Rodríguez (2013) indicated that the highest SO_2_ concentrations in SCT are recorded at stations downwind of the oil refinery at daytime under the inland breeze regime. González et al. (2011) determined that high SO_2_ concentrations daily recorded from 10 to 17 h in SCT are associated with inland sea breeze blowing caused by the entry of ship plumes in the city. Baldasano et al. (2014) determined that the refinery emissions play an important role in the SO_2_ exceedances of the legal limits; the area of influence of the refinery plume is local with a maximum radius of 3 km surrounding the facilities. Milford et al. (2008) developed a system for forecasting air pollution that predicts the probability of SO_2_ concentration exceeding certain thresholds for a measurement station located in SCT. Baldasano (2010) presented an air quality forecasting system for the Canary Islands, which makes a 48-h forecast of the main basic pollutants with a spatial resolution of 2 km (www.bsc.es/caliope/es).

Observed concentrations of NO_2_ in 2011 did not exceed EU limit values and the main emission source in SCT is road traffic (Baldasano et al. 2014). González and Rodríguez (2013) indicated that maximum NO concentrations are registered at traffic rush hours during working days, coinciding with low wind conditions, before building up of daylight breeze. NO dependence with wind direction is lower since vehicle exhaust emissions are a diffuse source. Reche et al. (2011) suggested that NO_2_ concentrations in SCT reach relatively low levels with respect to other sites (large cities in continental Europe) due to the good ventilation conditions on the island.

The three main sources of aerosols in SCT come from (1) anthropogenic emissions, (2) sea salt aerosol (SSA) due to its coastal character, and (3) mineral dust events from the Sahara. Rodríguez et al. (2008) manifested that the main anthropogenic sources of PM_10_ and PM_2.5_ in SCT come from road traffic and that photo-oxidation processes contribute significantly to the concentration of ultrafine particles (UFP). González et al. (2011) proposed that ship emissions may result in much higher concentrations of UFP than vehicle exhaust emissions. González and Rodríguez (2013) suggested that background levels of UFP are caused by traffic emissions and that elevated levels of photo-oxidation result from emissions from the port and the oil refinery. Reche et al. (2011) suggested that aerosol number concentration (N) increases at midday in SCT due to secondary formation of particles by means of photochemical nucleation processes from gaseous precursors as a consequence of the high solar radiation, growth of the mixing layer, increase in wind speed, and the consequent decrease of pollutant concentrations. Querol et al. (2008) determined that in SCT the mineral dust concentrations during African dust events (locally *calimas*) are much higher than in other regions of Spain because of its closeness to North Africa. Cordoba-Jabonero et al. (2011) characterized through the synergetic use of simultaneous remote sensing and in situ observations a dust intrusion plume from the Saharan region through observations at three stations (including SCT) along a common dust plume pathway. The vertical layering structure of those dust plumes was characterized, identifying different aerosol contributions depending on altitude. Dust layer top was found at 4.5–5 km height and in SCT backscatter profiling displayed a multi-layered structure through the overall atmosphere up to the top. Baldasano et al. (2014) determined that, at a synoptic level, particulate matter pollution is caused by episodes of Saharan dust intrusion with East synoptic winds (8.7% for the period 1998–2011), typical during the winter period. The SSA contribution is a subject that is not much studied in the Canary Islands. Spada et al. (2015) showed the importance of orography in its emission.

Cuevas et al. (2012) analyzed a 22-year surface O_3_ series at the subtropical high mountain Izaña station in Tenerife Island, assessing diurnal and seasonal O_3_ variations as well as trends. They found that higher O_3_ values were associated with air masses traveling above 4 km altitude from North America and North Atlantic Ocean, while low O_3_ was transported from the Saharan continental boundary layer (CBL). Aged air masses, in combination with sporadic inputs from the upper troposphere, are observed in spring, summer, and autumn. In summer time high O_3_ come from stratosphere-to-troposphere exchange processes in regions bordering the Canary Islands. Guerra et al. (2004) proposed that in urban areas such as Tenerife, O_3_ is mainly titrated by NO and replenished from the north due to the prevalence of NE trade winds. Downwind of urban areas, an ozone-excess (with respect to O_3_ levels in the oceanic boundary layer) is frequently recorded due to photochemical formation in aged air. Reche et al. (2011) observed highest daily O_3_ values in spring and in the first half of summer time and suggest that O_3_ daily patterns at SCT show levels at night similar to those registered at midday, a behavior induced by the continuous supply of fresh oceanic masses coupled with low local NO levels.

Baldasano et al. (2014) identified typical meteorological synoptic situations in SCT for the period 1998–2011. The dominant situation is the trade winds (NE component), which in SCT represent 28.8% of the situations and create local recirculation processes due to the orography with 31.9% of the situations. The NW winds are present in a 9.6% and give rise to recirculations in a 15.1%. The East component is given in 8.7% and finally the direct winds of W only 5.7%. Besides, the urban scale transport of air pollutants in SCT is mainly driven by breeze circulation (Rodríguez et al. 2008). These recirculations, combined with the local daily wind patterns, create positive feedbacks that emphasize air pollution episodes.

The purpose of this study is to analyze the trends and patterns in the air quality of Santa Cruz de Tenerife during the period 2011–2015. Data from the extensive air quality monitoring network in SCT has been used in order to evaluate the evolution during that period of time. The study also corroborates that the high levels of SO_2_ are due to the refinery emissions and determines the temporal evolution of concentrations of NO_2_, PM_10_, PM_2.5_, and O_3_. It further checks the influence of weather conditions as well as obtaining temporal patterns of behavior of different pollutants.

## Materials and methods

### Study area

**Fig. 1:**
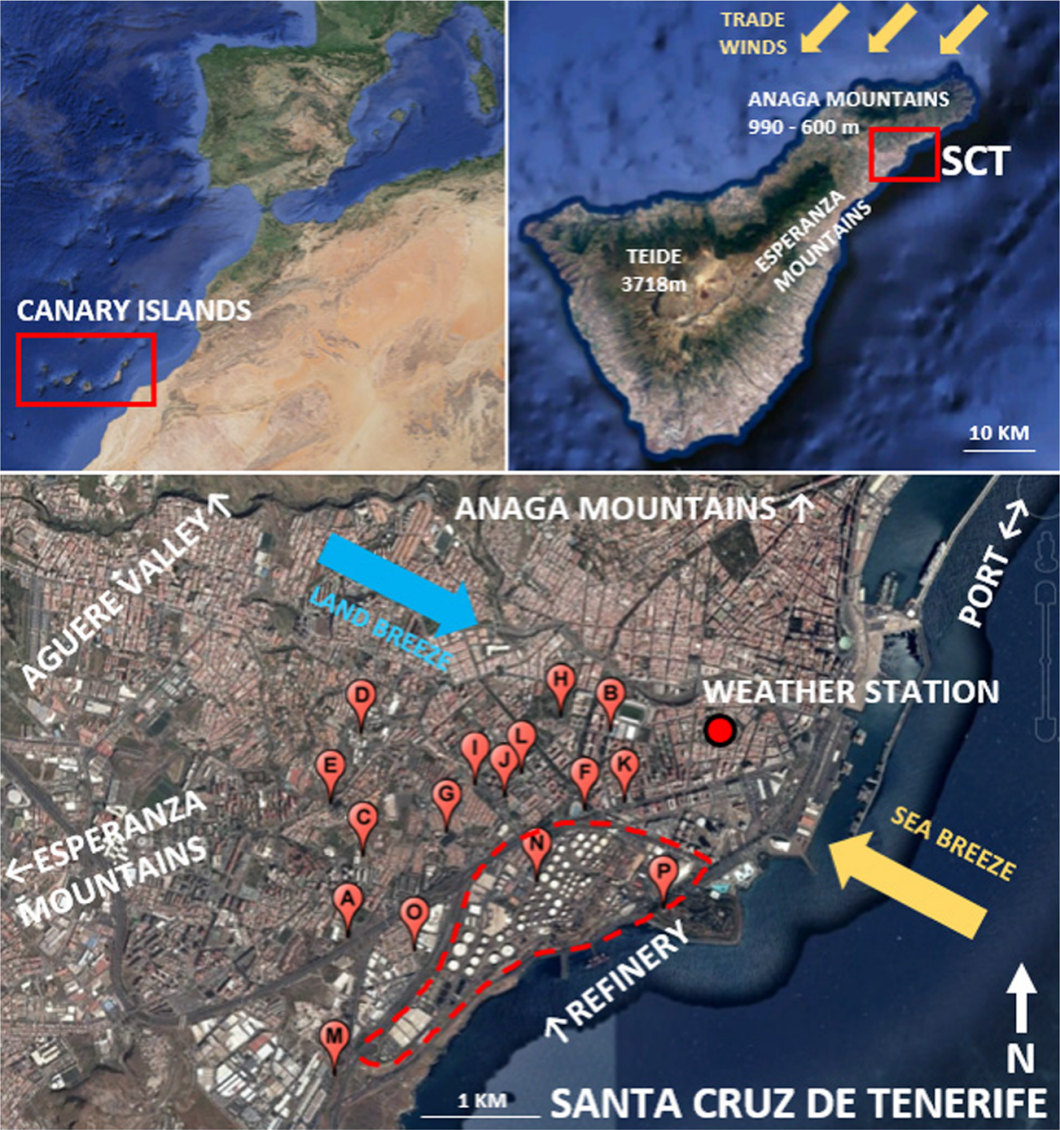
(*Top*) Location of the Canary Islands, main topographical features in Tenerife Island, and detail of the area of study, Santa Cruz de Tenerife. (*Bottom*) SCT map with stations and AEMET’s weather station C449C. See letter codes correspondence at Table S.1 in supplementary materials

Santa Cruz de Tenerife city (SCT) is a coastal city (Canary Islands: 28° 28′ N, 16° 15′ W; Fig. 1) with 204,000 inhabitants (INE 2016). The municipality is spread along 150 km^2^. The city lies at the foot of the Anaga mountain range (992 m, SW–NE ridge across the island) to the north. The ocean is to the east of the city, a uniform slope to San Cristóbal de la Laguna (Aguere Valley) limits the city to the northwest, and the Esperanza Mountains are situated in the southwest.

The prevailing winds at a synoptic scale are the trade winds that interact with the characteristic orographic features of the island (see Fig. 1), developing geographical effects in the lower layers of the atmosphere (Jorba et al. 2008; Baldasano et al. 2014). The local winds are characterized by daily cycles driven by typical sea-land breezes from coastal locations. These breezes have a strong influence on the transport of pollutants to the city (Rodríguez et al. 2008). The cold ocean currents present in the Tenerife Island moisten and cool down the surface air masses, creating thermal inversion at around 900–1200 m of height, impeding convective motions (Cuevas et al. 2012).

Furthermore, the proximity of the island to the African continent and the Azores anticyclone creates frequent mineral dust intrusions (the so-called Saharan episodes, and locally *calimas*) that have a strong impact on the air quality of SCT (Cordoba-Jabonero et al. 2011; Alonso-Perez et al. 2011a, b).

These orographic and meteorological characteristics in combination with anthropogenic emissions (oil refinery, road/maritime traffic) and Saharan dust intrusions create specific pollution episodes in the city.

The most significant air pollution anthropogenic sources in SCT are as follows:

- Road traffic. The city is crossed by busy roads: There is a highway that connects SCT to the southern part of the island (TF-1) and a fast track that connects La Laguna, the airport, and the northern part of the island with the city in the northwest direction (TF-5), with an average daily traffic (ADT) of more than 100,000 vehicles per day (CT 2016) (see Figs. 5 and 6). Vehicles with diesel cycle engines represent about 34% of the total vehicle fleet (being the commercial vehicles, buses and heavy duty trucks a 58% of the total diesel vehicles) (ICE 2016). The number of vehicles with diesel engine increased by 8% within the period 2011–2015 and diesel passenger cars increased by 20% (see Fig. S.1) (ICE 2016).
- Maritime traffic. It contributes with emissions due to ship incomings, outgoings and cruise hoteling that come from the eastern part of the city where the port is located. The global annual ship traffic at SCT port decreased by 9% within the time period in study (while merchant vessel traffic decreased by 17% and non-merchant vessel traffic increased by 38%) (see Fig. S.2) (APSC 2016).
- Oil refinery. The refinery is situated in the southeast along the coastline inside the metropolitan area of SCT (see Fig. 1). It has a refining capacity of 90,000 daily barrels (AOP 2010) and operated during 2011, 2012 and 2013 with some interruptions. In 2014 and 2015, it was not operative.

Baldasano et al. (2014) analyzed emission by pollutant and sector in the area of SCT (5 × 5 km^2^) in order to determine the contribution of each source to the total emissions. Road traffic was the main source of NO_*x*_ and PM with 57 and 61% of the total emissions respectively. The refinery was the main SO_*x*_ contributor (78%) and contributed with 40 and 25% of the total NO_*x*_ and PM emissions, respectively, in the area. The port activities emitted 12% of the SO_*x*_ and 13% of the PM.

### Air quality measurements

SCT has an air quality network of stations spread across an area of approximately 2.5 km^2^, concentrated in the urban area near the refinery and the port, shown in Fig. 1. Hourly data of SO_2_, NO_2_, PM_10_, PM_2.5_, and O_3_, which measured concentrations from the period 2011–2015 provided by the Canary Islands Government (GC 2016), has been processed, analyzed, and summarized.

Data quality objectives for air quality assessment are defined in the EU Directive 2008/50/EC (EC 2008) as a percentage of hourly data availability along the year. The minimum percentage of data capture for SO_2_, NO_2_, PM_10_, and PM_2.5_ is 90%. For O_3_, it is 75% for winter measurements and 90% for summer.

The active stations and pollutant data availability changed along the time period. Table S.1 in supplementary materials summarizes data availability and data quality per pollutant and station. The stations with more measured pollutants and better data quality are A, B, C, D, E, F, G, and H. The pollutants with more data availability are SO_2_ and O_3_. On the contrary, the available measurements of particulate matter are limited, especially PM_2.5_ data.

There are 7 stations (I, J, K, L, M, N, O, and P) with insufficient data capture within the time frame considered. Despite the minimum 90% of data quality objectives, in some cases, measurements from stations with a minimum annual data capture of 75% are used to achieve data continuation over time.

## Analysis and discussion

In this section, the air quality of SCT is assessed from a regulatory point of view, taking the EU Directive (EC 2008) and Spanish regulations (BOE 2011) into account for the period 2011–2015. Furthermore, air quality guidelines from the World Health Organization (WHO 2014) are also considered to evaluate air quality from a public health point of view. Weather patterns of the 2011–2015 period are compared to 1981–2010 averages to assess any possible influence in SCT air quality. Moreover, SO_2_ and NO_*x*_-O_3_ patterns are further analyzed.

### Sulfur dioxide (SO_2_)

The European air quality standards set limit concentration values for 1-h averages (350 μg/m^3^) not to be exceeded on more than 24 times per year and for 1-day averages (125 μg/m^3^) not to be exceeded more than 3 times per year. Furthermore, there is also an alert threshold value of 500 μg/m^3^ that, when exceeded over 3 consecutive hours, the authorities have to implement action plans to lower the levels of SO_2_. The WHO AQG is significantly stricter than the limit values set by the EU Directive (20 μg/m^3^ daily average).

Table 1 summarizes the information processed from stations which provide a minimum data of 90%. However, data from some stations with lower percentage of data capture are added to better assess concentrations evolution of this pollutant over time. See the table footnote for a description of the information shown.

**Table 1:**
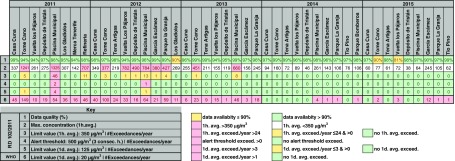
Air quality assessment of Santa Cruz de Tenerife 2011–2015 with regard to SO_2_ measured concentrations. Summary of maximum 1-h mean values measured, number of legal 1-h mean exceedances per year, number of alert thresholds reached per year, number of 1-day mean exceedances, and number of WHO AQG 1-day mean exceedances. EU air quality standards and WHO AQG for SO_2_ are also shown

In 2011 Refineria, Tome Cano and Piscina Municipal stations registered 1-h mean concentrations above 350 μg/m^3^. Piscina Municipal, exceeded 46 times the 1-h mean limit value, practically duplicating the legal 24 exceedances per year.

Tome Cano and Piscina Municipal measured daily mean concentrations above 125 μg/m^3^. Piscina Municipal station exceeded 4 times the legal 3 daily mean limit exceedances per year. Additionally, these measuring exceeded concentrations of 1000 μg/m^3^ on more than one occasion during the year. Furthermore, this measuring station registered two exceedances of the alert threshold value (more than 500 μg/m^3^ during 3 consecutive hours).

**Fig. 2:**
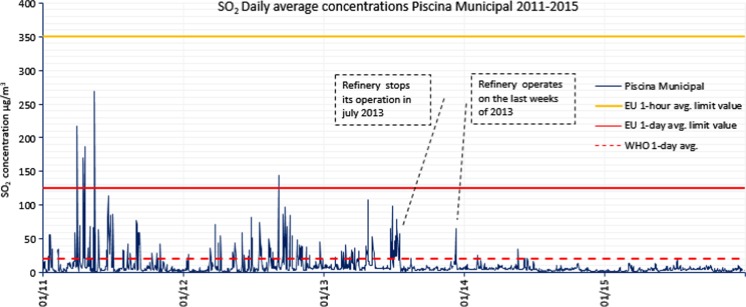
SO_2_ daily mean concentrations registered in Piscina Municipal over 2011–2015. Hourly and daily mean limit values from the EU Directive and daily mean WHO AQG values are also shown

In 2012, more exceedances of the 1-h limit value were registered, although none of them reached the legal 24 exceedances per year, showing a trend towards diminishing SO_2_ concentrations (see Fig. 2). Piscina Municipal station registered 13 times values higher than 350 μg/m^3^, reached the alert threshold once, and exceeded the 1-day limit value once.

During 2013, only Piscina Municipal and Tome Cano stations exceeded the 1-h mean limit value and none of them exceeded neither the alert threshold nor the 1-day mean limit value.

In 2014 and 2015, none of the stations exceeded any of the legal limit values.

However, SO_2_ concentrations measured at all the stations were occasionally harmful to public health according to daily means WHO AQG during 2011, 2012, and 2013. Tome Cano and Piscina Municipal stations registered the largest number of days exceeding these values and reaching up to almost 150 and 55 days, respectively, in 2011 and 100 and 65 days in 2012. In 2014 and 2015, WHO AQG exceedances were significantly lower: 4 days in Piscina Municipal station in 2014 and 12 days in García Escámez station in 2015.

Figure 2 shows the trend over time of SO_2_ daily average concentrations measured in Piscina Municipal station, the one that measured the highest concentrations and the largest number of exceedances of legal values registered within 2011–2015. It can be observed that measured concentrations in 2011, 2012, and the first half of 2013 were significantly high with several daily mean legal exceedances and occasionally harmful to public health (daily means >20 μg/m^3^), coinciding with the active periods of the refinery. In the last weeks of 2013, the refinery did not operate continuously.

Yearly averages of Tome Cano, Piscina Municipal, and Casa Cuna fluctuated between 9.3 and 20.4 μg/m^3^, (mean 13.5 μg/m3) during 2011–2012. The background SO_2_ concentrations (calculated as 1-year averages of daily means) during 2014 and 2015 (refinery not operative) were within a range of 2.5–7.1 μg/m^3^ (mean 4.5 μg/m^3^), far below the WHO AQG (daily mean 20 μg/m^3^). It is then confirmed that the refinery plays an important role in SO_2_ concentration levels in the city as Baldasano et al. (2014) stated.

**Fig. 3:**
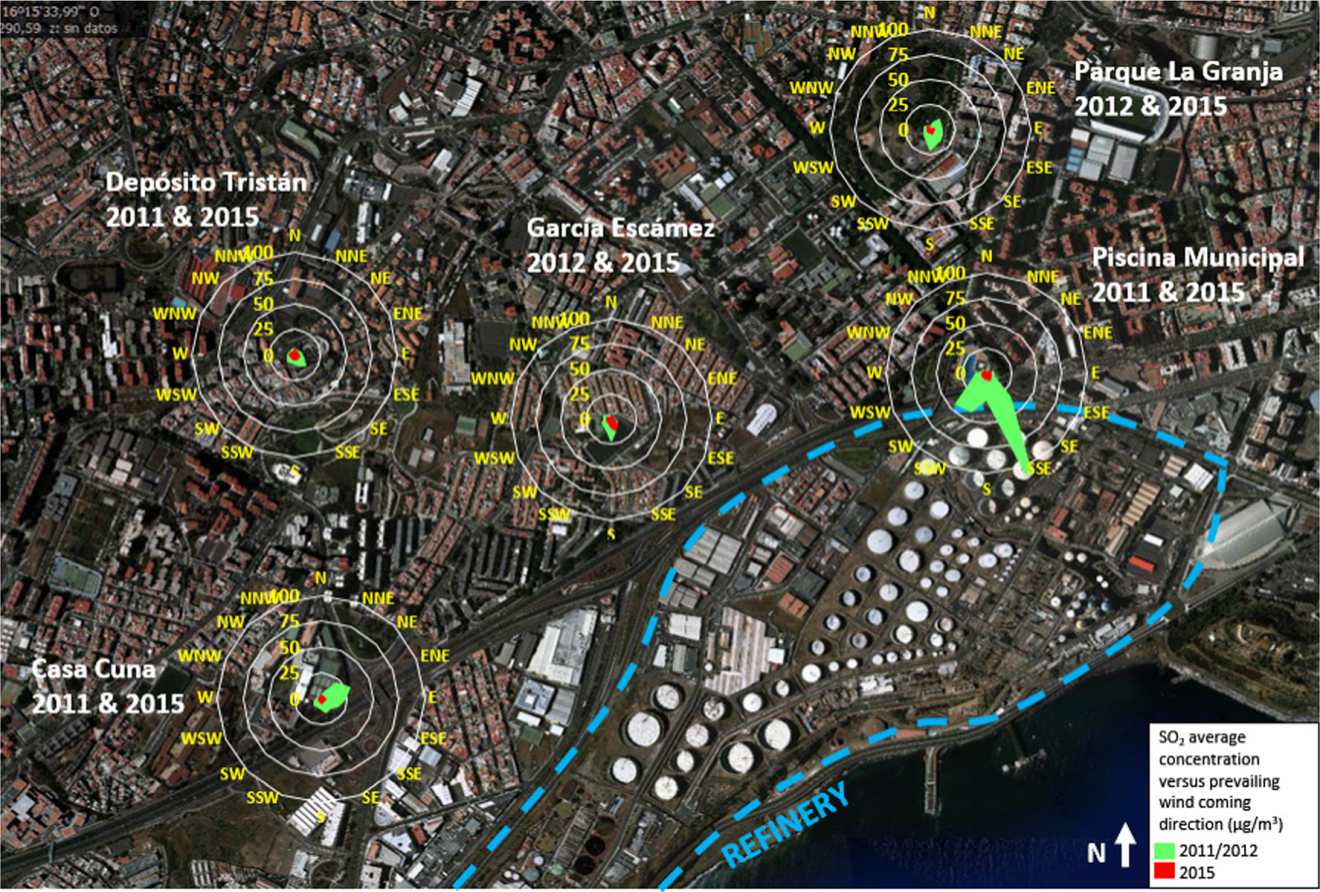
Average SO_2_ concentration (μg/m^3^) radar plots with respect to wind prevailing directions. *Green concentration plots* when the refinery was in operation and *red plots* when the refinery was inactive. The radar plots are located at the stations’ corresponding position in the city

Figure 3 shows mean SO_2_ concentrations with respect to the prevailing wind’s coming directions. Each radial chart represents a different measuring station positioned at its corresponding location in SCT.

Each chart is divided into 16 wind directions. The radial axis corresponds to measured SO_2_ concentrations. Hourly mean concentrations were grouped into wind directions, averaged and plotted to each chart per corresponding year. There are two plots per radial chart, the green one with concentrations measured during the operation of the refinery (years 2011 or 2012, depending on the available data) and the red one when the refinery did not operate (2015), showing significant differences in concentrations values and in direction trends for the different time periods. The highest mean concentrations during 2011 or 2012 were registered in prevailing wind directions that point directly to the refinery stacks, confirming the main source of this pollutant (corroborating González and Rodríguez 2013). However, the mean concentrations registered in 2015 were radically lower and did not show so clear prevailing wind direction as in 2011–2012.

When the refinery was not in operation (years 2014 and 2015), all the SO_2_ measured concentrations met the legal European air quality standards. The rest of SO_2_ sources (mainly the port activities) contributed to a background concentration of 4.5 μg/m^3^, not harmful to public health according to the WHO AQG. Figures S.3, S.4, S.5, and S.6 in supplementary materials show SO_2_ concentrations over 2011–2015 measured by other stations in SCT.

### Nitrogen dioxide (NO_2_)

The EU air quality standards set annual and hourly mean limit values for the protection of human health. The limit value for the annual mean NO_2_ concentration is 40 μg/m^3^. The 1-h limit value threshold is 200 μg/m^3^ and can be exceeded on up to 18 days per year. There is also defined an “alert” threshold value of 400 μg/m^3^ which, when exceeded over three consecutive hours, compel authorities to implement short-term actions. The threshold values used in the human health objectives set by the EU Directive are identical to the WHO AQG for NO_2_.

Table 2 summarizes the information processed, which provides a minimum data capture of 90%. However, data from some stations with lower percentage of data capture are added to better assess concentrations evolution of this pollutant over time. See the table footnote for a description of the information shown.

**Table 2:**
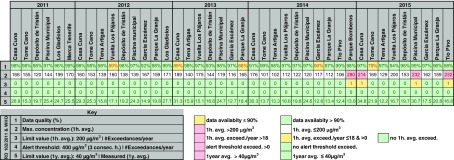
Air quality assessment of Santa Cruz de Tenerife 2011–2015 with regard to NO_2_ measured concentrations. Summary of maximum 1-h mean values measured, number of legal 1-h mean exceedances, number of alert thresholds reached, and number of WHO AQG 1-year mean exceedances. The EU air quality standards and the WHO AQG for NO_2_ are also shown

No EU air quality standards (nor WHO AQG) were exceeded during 2011, 2012, and 2013 in any of the air quality stations. One station exceeded on one occasion the 200 μg/m^3^ hourly mean limit value in 2014 and three in 2015, far below from the legal 18 maximum exceedances allowed per year.

**Fig. 4:**
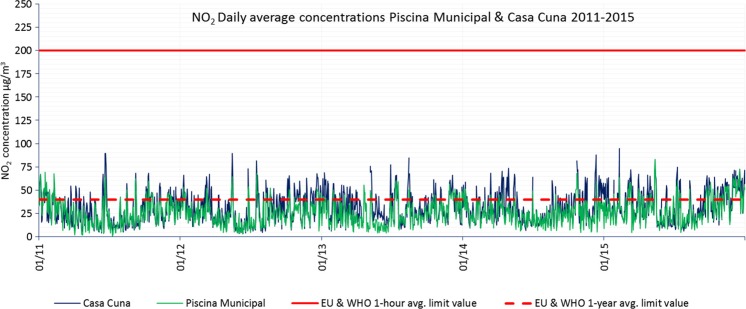
NO_2_ daily mean concentrations registered in Piscina Municipal and Casa Cuna during 2011–2015. Daily mean and annual mean limit values are shown. The value limits from EU air quality standards and WHO AQG are the same

Figure 4 shows daily mean concentrations measured in stations Piscina Municipal and Casa Cuna with higher NO_2_ annual mean concentrations during 2011–2015. The higher concentrations are attributable to the proximity of these stations to the busiest roads in the city (see Fig. 6).

**Fig. 5:**
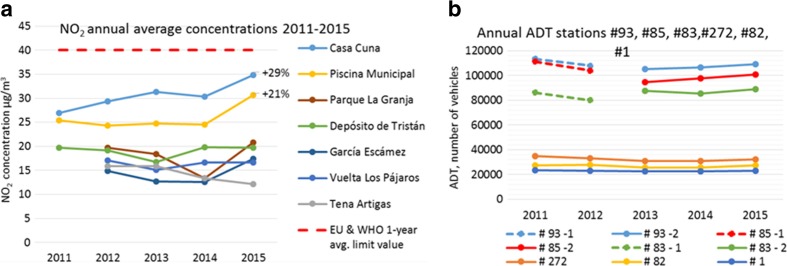
**a** NO_2_ annual mean concentrations registered in some stations during 2011–2015. The percentages show the NO_2_ concentration increment from 2011 to 2015. The value limits from EU air quality standards and the WHO AQG are the same. **b** Average daily traffic measured by traffic gauge stations (see Fig. 6) per year

A clear trend of annual frequency can be observed during 2011, 2012, 2013. and 2014 where NO_2_ concentrations reach lower values during summer and higher values during winter, although high episodic values are given in summer. From 2015, the peak concentration values seem higher than in the previous years in accordance with Fig. 5a, which shows a significant non-uniform annual mean concentration build-up: from 25 to 31 μg/m^3^ (+21%) in Piscina Municipal and from 27 to 35 μg/m^3^ (+29%) in Casa Cuna from 2011 to 2015.

**Fig. 6:**
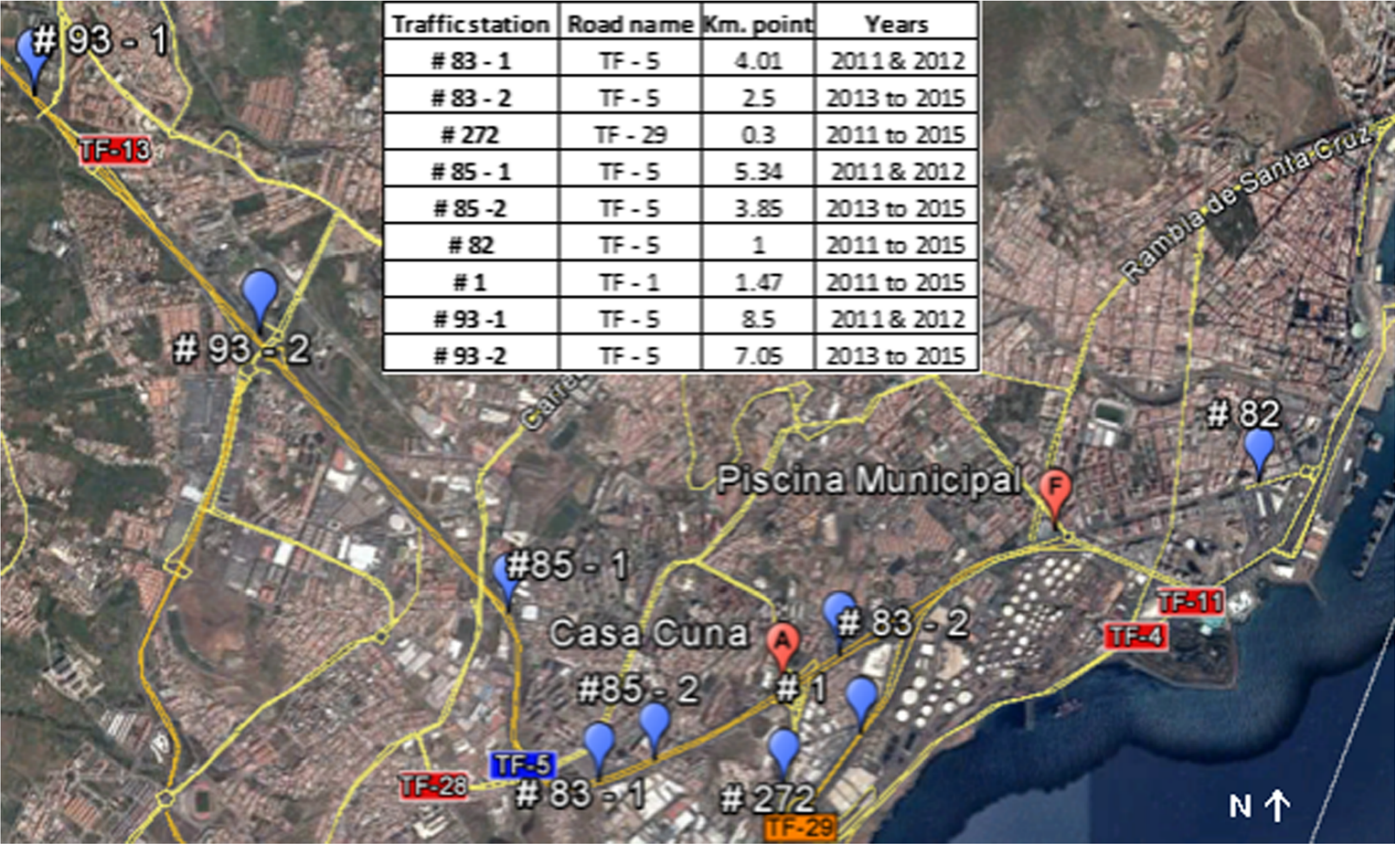
Traffic gauge stations positions and Casa Cuna and Piscina Municipal measurement stations. The table shows the kilometric points in the road over the years. Stations #93 and #83 changed their position in 2013

Figure 6 shows the positions of traffic measurement stations in high-traffic routes near Casa Cuna and Piscina Municipal air quality stations. Figure 5b shows the average daily traffic (ADT) evolution between 2011 and 2015 on the stations shown in Fig. 6. As a general trend, there was a slight drop in ADT from 2011, marking a minimum in 2013 and a smooth increase until 2015. However, traffic at all stations was slightly lower in 2015 than in 2011. Stations #83, #85, and #93 changed their position in 2013 without affecting the overall conclusions of the analysis.

Figure S.2 shows the evolution of maritime traffic in the port of SCT between 2011 and 2015. The traffic of merchant ships decreased considerably (−17%) throughout the time series. Non-merchant ship traffic, which accounts for only a third of the total port traffic, increased by 38% between 2011 and 2015. Thus, as a global trend, there was a 9% decline in sea traffic in the SCT port from 2011 to 2015.

Figure S.1 shows the evolution of the vehicular composition of the province along the time series. The number of vehicles with gasoline engine decreased, specifically by 1.2% in passenger cars and by 12.5% in trucks and commercial vehicles. In the case of diesel cycle vehicles, the number of trucks and commercial vehicles remained stable (+0.2%). However, it is noteworthy that there was a 20% increase in diesel passenger cars, showing a constant dieselization of the vehicle fleet in the region during 2011–2015.

The decrease in road traffic and sea traffic in the city in the period 2011–2015 and the significant change in the passenger car engine type composition suggest that the increase in NO_2_ concentrations, especially in the stations of Piscina Municipal and Casa Cuna, was due to the progressive dieselization of the vehicular fleet of the province.

### Particulate matter (PM_10_ and PM_2.5_)

The EU Directive sets limit values for daily and annual PM_10_ concentrations, while values for only annual PM_2.5_ concentrations have been set. The daily average concentration value for PM_10_ is set at 50 μg/m^3^ not to be exceeded more than 35 days per year. The annual PM_10_ limit value is 40 μg/m^3^. The deadline for meeting the exposure concentration of 25 μg/m^3^ obligation for PM_2.5_ is 2015. The WHO AQG regarding maximum annual mean concentrations are significantly stricter than the EU air quality standards (20 μg/m^3^ for PM_10_ and 10 μg/m^3^ for PM_2.5_).

The EU Directive provides the member states with the possibility of subtracting the contribution from natural sources when limits are exceeded before comparing the ambient air pollutant concentrations to the limit values (EC 2011). In this assessment, a methodology for application in Spain (Querol et al. 2006, 2010) is used to determine the contribution from natural sources to the levels of observed particulate matter concentrations. The Ministerio de Agricultura y Pesca, Alimentación y Medio Ambiente (MAPAMA 2016), provides natural sources contribution values to be subtracted to measured concentrations (PM_10_ data for 2011–2015 and PM_2.5_ data only for 2015).

#### PM_10_

Table 3 summarizes the information processed which provides a minimum data capture of 90%. However, data from stations with more than 75% of valid data over the year are added to better assess concentration evolution of this pollutant over time. See the table footnote for a description of the information shown.

**Table 3:**
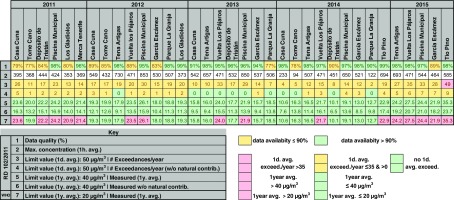
Air quality assessment of Santa Cruz de Tenerife 2011–2015 with regard to PM_10_ measured concentrations. Summary of maximum 1-h mean values measured, number of 1-day mean exceedances per year with and without particulate matter natural sources contribution, 1-year mean measurements with and without particulate matter natural contribution and WHO AQG 1-year mean exceedances. The EU air quality standards and the WHO AQG for PM_10_ are also shown

All the stations registered daily mean exceedances within the time period in the study. In 2015, Vuelta los Pájaros reached the legal maximum 35 days above the limit concentration and Tio Pino registered 49 days of exceedances, 14 days above the legal maximum. However, subtracting the contribution of the natural sources, these stations lowered the number of exceedances to 6 and 9 days, respectively, both within the legal limit values. None of the stations reached the EU annual mean limit values (40 μg/m^3^).

The possibility of subtracting natural sources contributions before comparing the air pollutant concentrations with the limit values does not mean that pollutants of natural origin are not adversely affecting health (EC 2011). However, all the stations in 2011 and 2015 measured annual mean values above the stricter WHO AQG (20 μg/m^3^) with a measured maximum annual mean concentration of 35 μg/m^3^ in Tio Pino in 2015. In 2012 and 2013, Vuelta los Pájaros, and Piscina Municipal exceeded the WHO AQG annual mean and Vuelta los Pájaros, and Tio Pino in 2014.

**Fig. 7:**
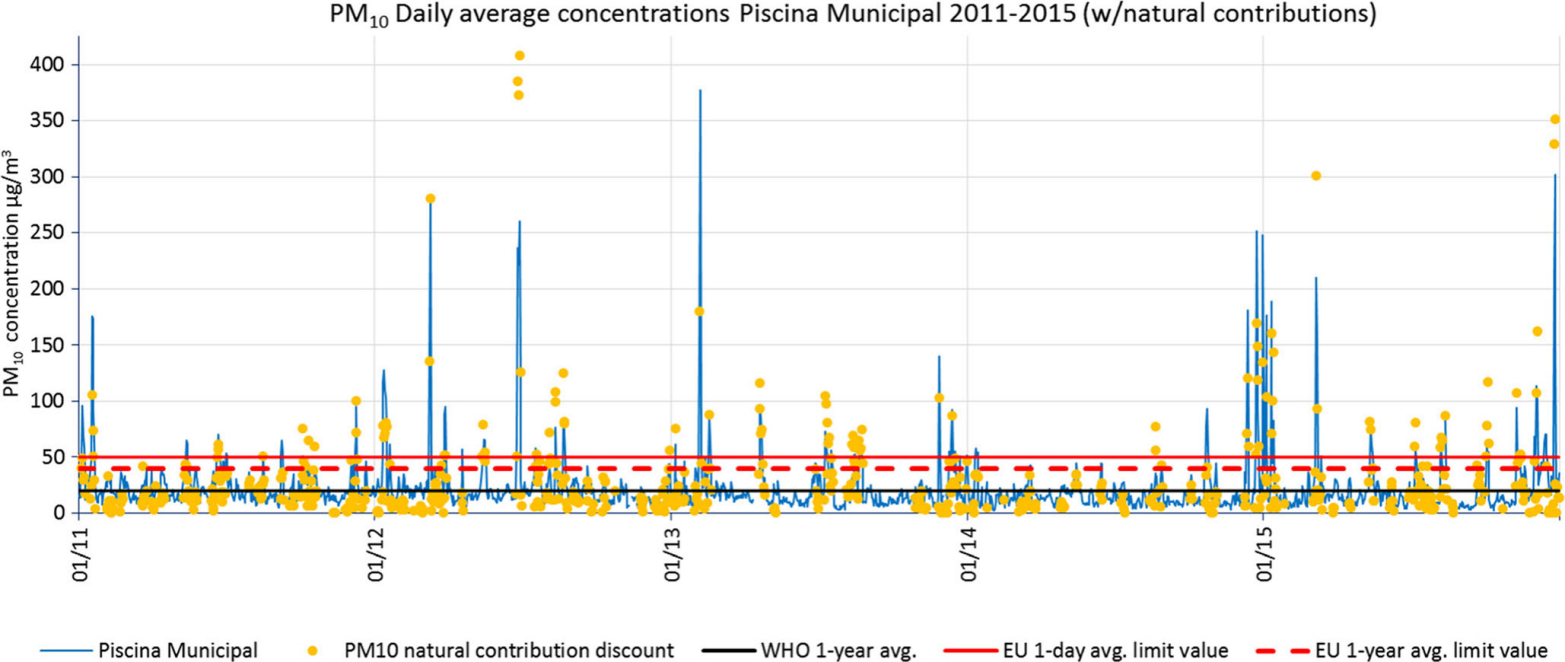
PM_10_ daily mean concentrations registered in Piscina Municipal measuring station during 2011–2015. Daily and annual mean limit values from the EU Directive and from the WHO AQG are also shown. *Yellow dots* are Saharan dust contributions provided by MAPAMA to be subtracted to measured concentrations through the methodology proposed by Querol et al. (2006, 2010)

Figure 7 shows daily mean concentrations measured in Piscina Municipal station over the time period in the study. The yellow dots are Saharan dust contribution to PM_10_ levels in SCT (from MAPAMA 2016). An annual average of 120 days of Saharan intrusion occurred in SCT in the period 2011–2015. A high correlation between exceedances of daily mean legal concentrations and Saharan intrusion episodes can be observed.

**Fig. 8:**
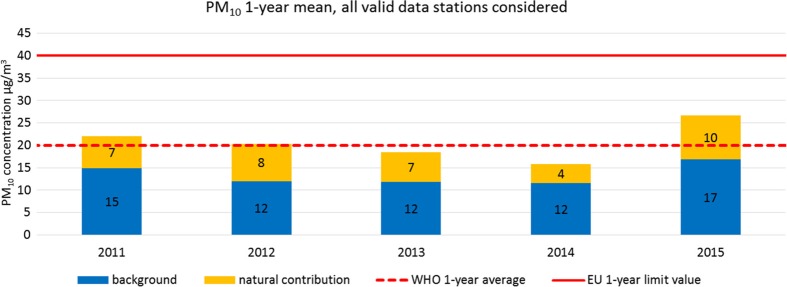
Average of PM_10_ 1-year mean concentrations from all stations with more than 75% of valid data during 2011–2015

Figure 8 shows the average of PM_10_ annual mean concentrations registered at all stations per year. The background levels fluctuated between 12 and 17 μg/m^3^ (average 13.5 μg/m^3^). In 2012, 2013, and 2014, the annual mean PM_10_ concentration remained below the WHO AQG due to a nearly constant lower background level of 12 μg/m^3^ and low natural sources contributions. The concentrations measured in 2011 and 2015 exceeded WHO AQG. The year 2015 was the worst in terms of public health air quality due to a significantly higher background level of 17 μg/m^3^ that, added to the highest level of natural sources contribution (10 μg/m^3^) in the period made an average annual concentration of 27 μg/m^3^, above the WHO 1-year mean AQG (20 μg/m^3^).

Figures S.7, S.8, and S.9 in supplementary materials show PM_10_ concentrations over 2012–2015 measured by other SCT stations.

#### PM_2.5_

Data availability for PM_2.5_ is poor: in 2011, none of the stations reached 90% of data capture and in 2012 only 2 stations reached this percentage. Table 4 shows data from stations with a minimum data capture of 75% (see the table footnote for a description of the information shown). There were no exceedances of the annual average concentration (25 μg/m^3^) determined by EU air quality standards in any of the stations over the time series. WHO annual mean AQG were met during 2011–2014. However, in 2015, three stations exceeded WHO annual mean AQG (during 2015 the annual mean concentrations approximately doubled with respect to 2014 in all the stations). Almost all the stations registered exceedances of WHO daily mean AQG during the whole period. The number of daily mean exceedances in 2015 was about 3 times the exceedances in 2014. The PM_2.5_ annual mean concentrations during 2011–2015 ranged between 3.7 and 12.3 μg/m^3^ (mean 7.9 μg/m^3^).

**Table 4:**
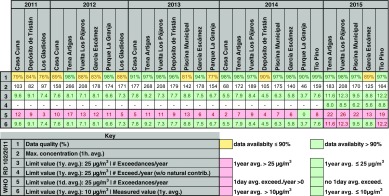
Air quality assessment of Santa Cruz de Tenerife 2011–2015 with regard to PM_2.5_ measured concentrations. Summary of maximum 1-h mean values measured, 1-year mean measurements (with the subtraction of natural sources contribution only in 2015, provided by MAPAMA), and comparison with the WHO AQG 1-year mean. The EU air quality standards and the WHO AQG for PM_2.5_ are also shown

MAPAMA only provides data of natural sources contribution to the PM_2.5_ levels from the year 2015. This contribution represents an approximate value of 3.5 μg/m^3^, about one-third of the measured concentrations.

**Fig. 9:**
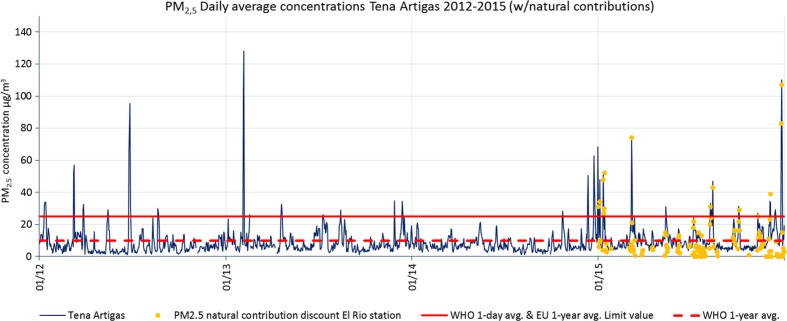
PM_2.5_ daily mean concentrations registered in Tena Artigas measuring station during 2012–2015. Annual mean limit values from the EU Directive and annual and daily mean limit values from WHO AQG are also shown. From 2015, *yellow dots* represent the natural source contributions calculated provided by MAPAMA to be subtracted to measured concentrations through the methodology proposed by Querol et al. (2006, 2010)

Figure 9 shows daily mean PM_2.5_ concentrations measured in Tena Artigas station over 2012–2015. The yellow dots are Saharan dust contributions to PM_2.5_ levels in SCT from the year 2015. A high correlation between exceedances of daily mean legal concentrations and Saharan intrusion episodes can be observed.

Figures S.10 and S.11 in supplementary materials show PM_2.5_ concentrations over 2012–2015 measured by other SCT stations.

### Ozone (O_3_)

EU air quality standards set for health protection specify a maximum daily 8-h mean target value is 120 μg/m^3^, not to be exceeded more than 25 days per year in a 3-year average starting from 2010. The long-term objective is no exceedance of the target value at all. There is also a “public information” threshold (180 μg/m^3^, hourly mean) and an “alert” threshold (240 μg/m^3^, hourly mean) for health protection. When the public information threshold is exceeded, the authorities have to notify the citizenship. When the alert threshold is exceeded for three consecutive hours, the authorities are required to set up a short-term action plan. The WHO AQG for O_3_ sets a stricter daily maximum 8-h mean concentration of 100 μg/m^3^.

Table 5 summarizes the information processed, which provides a minimum data capture of 90%. See the table footnote for a description of the information shown.

**Table 5:**
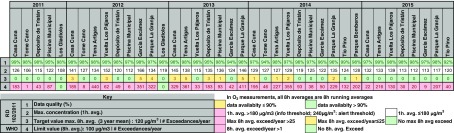
Air quality assessment of Santa Cruz de Tenerife 2011–2015 with regard to O_3_ measured concentrations. Maximum 1-h average concentrations, number of maximum daily 8-h mean values exceedances per year, and number of WHO AQG 8-h mean exceedances per year. The EU air quality standards and the WHO AQG for O_3_ are also shown

In 2011 and 2015, none of the stations measured maximum daily 8-h averages above the EU target values. However, in 2012, 2013, and 2014, Casa Cuna, Vuelta los Pájaros, and Parque la Granja were the stations that registered maximum daily 8-h averages above 120 μg/m^3^ but never reached the maximum of 25 days per year, determined as a 3-year average. When the stricter WHO AQG are taken into account, almost all stations exceeded sometimes the limits during the whole period.

**Fig. 10:**
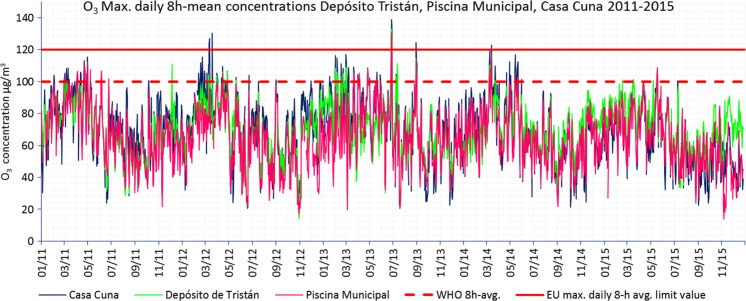
O_3_ maximum daily 8-h average concentrations registered in Depósito Tristán, Piscina Municipal, and Casa Cuna during 2011–2015. EU maximum daily 8-h mean target values from the EU Directive and maximum 8-h mean levels from WHO AQG are also shown

Figure 10 shows the evolution over time of the maximum daily 8-h mean concentrations from three stations that registered valid data during the whole period 2011–2015. A clear time pattern with annual frequency of ozone concentrations can be observed. Throughout the year, the highest observed concentrations (and therefore the exceedances of EU target values and WHO AQG) are given in February–March–April and the lowest values are given around August–September–October (see Fig. S.12 in supplementary materials). The intra-annual monthly average of maximum daily 8-h averages concentrations can vary up to 35% in March with respect to September.

### Weather patterns

The temperature, rainfall, and relative humidity registered in AEMET’s SCT weather station (C449C, see Fig. 1 for location) during 2011–2015 were analyzed and compared to average weather for the period 1981–2010 to determine any significant variations that could have had any influence on SCT air quality.

The monthly mean temperatures registered in January and February of 2012, 2014 and 2015 were between 1.2–2 °C lower than the average of 1981–2010. The years 2011, 2012, and 2015 registered less rainfall than the 1981–2010 period (−23, −26, and −6%, respectively). On the contrary, the years 2013 and 2014 registered 39 and 89% of more rainfall, respectively. This fact could have an influence on the lower 2013 and 2014 PM_10_ yearly average concentration (see Fig. 8) due to a washout effect. No significant changes in relative humidity patterns were registered during 2011–2015 with respect to 1981–2010 weather patterns.

The wind data from 2011 to 2015 indicate a clear process of sea-land breezes, dominated by the E-ENE (22–23% of situations) vs. W-WNW (21%) directions. The wind intensity was higher in 2014 and 2015 compared to 2011–2013 (winds over 4 m/s on 5.2 and 14.4% of situations, respectively). The existence of an S component with a frequency between 7 and 8% is significant, and the non-existence of N component is due to the orographic situation of SCT (see wind roses in Fig. S.13, supplementary materials).

### SO_2_ concentrations matrix

Figures S.14 and S.15 in supplementary materials show average hourly SO_2_ concentrations per month during the period 2011–2015 for Casa Cuna and Piscina Municipal stations. The highest concentrations were registered in 2011, 2012, and 2013 during the refinery active period.

SO_2_ levels measured at Piscina Municipal exhibit a high dependence on the local wind conditions due to the proximity to the refinery. Substantially higher SO_2_ concentrations and their variability throughout the day were registered. The maximum SO_2_ levels were measured between 10 and 16 h when the breeze generates the highest wind speeds and directs the refinery plumes to the air quality measuring station. The peak SO_2_ levels in Casa Cuna were significantly lower than in Piscina Municipal; besides, concentration daily variability was lower (creating slightly higher values during early morning and late afternoon to night) due to the distance to emission point. The SO_2_ concentrations throughout the year were more variable in Piscina Municipal, with the highest levels registered from March to September in 2011 and 2012. From July 2013, the refinery stopped its activity causing the SO_2_ levels to fall sharply. The background SO_2_ levels created by the port activities were higher during the daytime in 2014 and 2015. The lowest values were registered in January and February.

### Analysis of NO_*x*_-O_3_ cycles

**Fig. 11:**
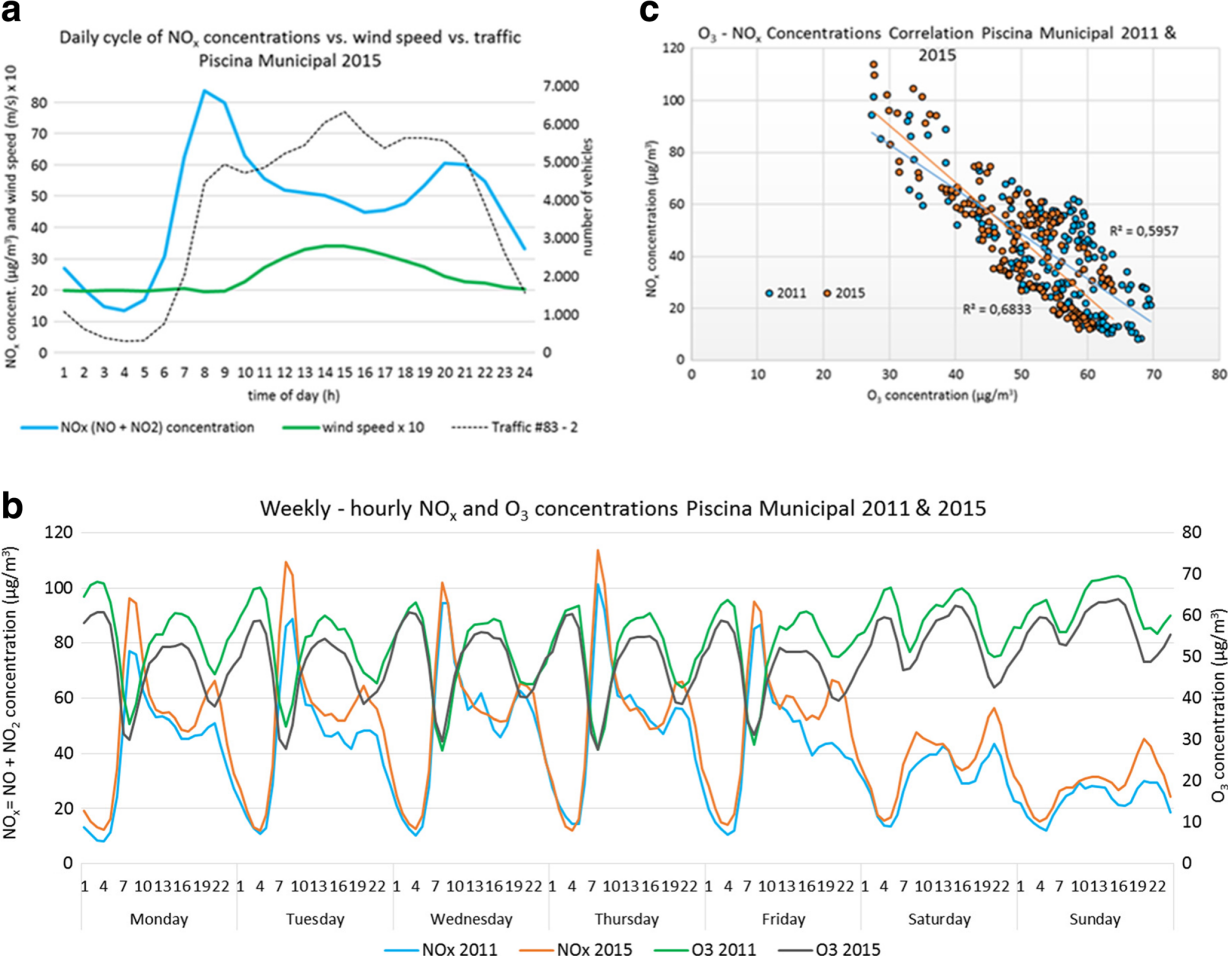
**a** Daily cycle of average NO_*x*_ concentrations measured at Piscina Municipal, average of wind speed (multiplied by 10 for a matter of visualization), and average of number of vehicles in traffic station #83-2 (see Fig. 6 for city location) for 2015. **b** Weekly-hourly cycles of average O_3_ and NO_*x*_ concentrations measured at Piscina Municipal for 2011 and 2015. **c** Dispersion diagram of weekly-hourly average concentrations of O_3_ and NO_*x*_ measured at Piscina Municipal for 2011 and 2015

Figure 11a shows the mean daily cycle of NO_*x*_ measured at Piscina Municipal, wind speed average (multiplied by 10 to improve visualization) and ADT measured by traffic station #83-2 (see Fig. 6 for location) for the year 2015. The traffic is minimum at late night (4-5 h) with three peaks (8–9, 14–16, and 19 h). The maximum daily NO_*x*_ concentrations were registered at 8–9 h, not when the traffic is highest but when the local wind speed reaches its daily minimum (∼2 m/s), hindering pollutants’ dispersion in accordance with González and Rodríguez (2013). Later, the sea breeze progressively builds up reaching its maximum speed between 13 and 16 h (∼3–4 m/s). At this time, the traffic is the busiest but the breeze disperses pollutants so NO_*x*_ levels register a relative minimum. The second maximum of NO_*x*_ concentrations is measured at 20–21 h, when the local wind speed descends and the traffic reaches its evening peak. Figure 11b shows the weekly cycle of average O_3_ and NO_*x*_ concentrations measured at Piscina Municipal for the years 2011 and 2015. On working days, NO_*x*_ concentrations followed a clear daily cycle with two maximum values at 8 and 20–21 h. At weekends, NO_*x*_ concentrations were lower and showed differences with lower daily variability and with two relative highs recorded at noon and at night. This is attributable to the lower road traffic on weekends (station #83 year 2011: ADT working days, ∼106,200 vehicles/day; ADT Saturdays, ∼77,000 vehicles/day; ADT Sundays, ∼57,900 vehicles/day). The minimum daily concentrations measured on weekends were comparable to those during the rest of the week.

The O_3_ weekly cycle shows a clear “weekend effect” where the O_3_ levels are significantly higher and exhibit lower variability during the weekend because of the lower and less variable NO_*x*_ levels present during weekend days.

NO_*x*_ concentrations measured in Piscina Municipal were higher in 2015 (∼+21%, annual mean) with respect to 2011, which explains O_3_ lower concentrations in 2015 (∼−16%, annual mean) with respect to 2011.

Figure 11 **c** shows the correlation between average weekly-hourly concentrations of O_3_ and NO_*x*_. O_3_ levels exhibit daily concentration cycles well anti-correlated with NO_*x*_ concentration (*R* ^2^ = 0.68 in 2011 and *R* ^2^ = 0.59 in 2015; *n* = 168). This significant anti-correlation shows that O_3_ is mainly titrated by locally emitted NO during traffic rush hours as proposed by Guerra et al. (2004). The O_3_ daily cycle presents two peaks, one during diurnal hours due to photochemical production and the other at night induced by the supply of fresh oceanic air masses and low local NO levels as suggested by Guerra et al. (2004) and Reche et al. (2011).

Figures S.16 and S.17 in supplementary materials show average hourly NO_2_ concentrations per month during the period 2011–2015 for Casa Cuna and Piscina Municipal stations. The observed NO_2_ concentrations tended to increment during the period. NO_2_ levels followed the daily cycle described in Fig. 11a, where the minimum concentrations were registered at night and the maximum concentrations around 8–9 and 20–21 h. Summer months exhibited lower concentrations than winter months.

## Conclusions

Air quality trends and patterns in the coastal city of Santa Cruz de Tenerife (Canary Islands, Spain) for the period 2011–2015 were analyzed. SO_2_, NO_2_, PM_10_, PM_2.5_, and O_3_ pollutants measured by the air quality monitoring network were assessed from both a legal (compliance of EU Directive 2008/50/EC) and a public health point of view (exceedances of WHO guidelines).

The orographic and meteorological characteristics, the proximity to the African continent and the influence of the Azores anticyclone in combination with the anthropogenic (oil refinery, road/maritime traffic) and natural emissions create specific dispersion conditions.

The refinery was the primary source of SO_2_. EU SO_2_ hourly limit values (350 μg/m^3^) were exceeded 46 times in Piscina Municipal station (maximum legal 24 per year) and daily average limit values (125 μg/m^3^) were exceeded 4 times (maximum legal 3 per year) in 2011 and alert thresholds were reached twice in 2011 and once in 2012 in the same station. The WHO daily mean AQG (20 μg/m^3^) were occasionally exceeded during 2011, 2012, and 2013 in all the stations, especially Tome Cano, Piscina Municipal, and Casa Cuna, with annual averages in 2011 and 2012 between 9.3 and 20.4 μg/m^3^. The spatial analysis of SO_2_ concentrations with respect to prevailing winds corroborate a clear influence of the refinery to the SO_2_ levels. In 2014 and 2015, the refinery did not operate and the concentrations fell abruptly to background levels of 2.5–7.1 μg/m^3^ far below from the WHO AQG. The influence of port emissions to the SO_2_ levels in the city is limited.

SCT complied with NO_2_ EU limit values (1-h average 200 μg/m^3^, 1-year average 40 μg/m^3^) as well as the WHO AQG (same values as EU air quality standards) for the period 2011–2015. However, the annual mean concentrations increased in a non-uniform way among stations: 25 μg/m^3^ to 31 μg/m^3^ (+21%) in Piscina Municipal and 27 μg/m^3^ to 35 μg/m^3^ (+29%) in Casa Cuna from 2011 to 2015 due to a progressive dieselization of the vehicle fleet of the province (diesel-powered passenger cars increased by 20% from 2011 to 2015).

Regarding PM_10_, only two air quality stations reached the 35 limit exceedances of daily EU limit values (50 μg/m^3^) before subtracting natural contributions (EC 2011) during 2011–2015 (Vuelta Los Pájaros, 35 exceedances; Tio Pino, 49 exceedances). After the subtraction, none of the stations reached the legal limit of 35 exceedances. Nevertheless, all the stations occasionally registered daily mean PM_10_ concentrations above 50 μg/m^3^ during 2011–2015. None of the stations exceeded the annual mean EU limit values (40 μg/m^3^). However, all the stations in 2011 and 2015 exceeded the annual mean WHO AQG (20 μg/m^3^), and many stations did in 2012–2014. Observed PM_10_ annual average concentrations in all the stations fluctuated between 10.1 and 35.3 μg/m^3^ (mean 20 μg/m^3^), where background concentrations were 6.5 to 24.4 μg/m^3^ with a natural contribution of 4.2 to 9.1 μg/m^3^. PM_10_ annual mean levels were the lowest during the most rainy years (2013 and 2014), suggesting a washout effect.

None of the stations reached the PM_2.5_ annual mean EU target values (25 μg/m^3^, limit values valid from 2015 onwards). However, almost all the stations registered exceedances of WHO daily mean AQG (25 μg/m^3^). The WHO annual mean AQG (10 μg/m^3^) was only exceeded during 2015 in three of the five stations (Tena Artigas, Vuelta Los Pájaros, Tio Pino). Annual average concentrations for all the stations during 2011–2014 ranged between 3.7 and 9.6 μg/m^3^ (mean 7.3 μg/m^3^). Data for PM_2.5_ natural contributions is available only for 2015, and the observed concentrations during 2015 were significantly higher than the previous years (approximately doubled from 2014) and fluctuated between 8.8 and 12.3 μg/m^3^, where background concentrations were 5.6 and 8.8 μg/m^3^ with natural contribution 3.2–3.7 μg/m^3^.

SCT complied with the EU target values for O_3_ (maximum legal 25 per year as a 3-year average of 120 μg/m^3^, maximum daily 8-h mean). However, stricter WHO AQG (100 μg/m^3^, 8-h mean) were sometimes exceeded in all the stations for the whole time period. The O_3_ levels showed a marked tendency of annual frequency where the highest values were registered in February–March-April and the lowest in August–September–October. A significant anti-correlation between O_3_ and NO_*x*_ concentrations was identified suggesting that O_3_ is primarily titrated by locally emitted NO. O_3_ exhibited a clear “weekend effect” with higher levels and lower variability during the weekend because of the lower and less variable NO_*x*_ levels.

The air quality monitoring network in SCT is dense. Too many stations are positioned in a too small area giving poor spatial information. The information provided for SO_2_, NO_2_, and O_3_ is correct, perhaps even excessive. However, the particulate matter data provided by the network is insufficient. It is necessary an efficiency improvement of the network (fewer stations more spread across the region, providing better data quality) to better assess the air quality in the city.

The EU air quality standards are not sufficiently restrictive in comparison to the WHO AQG for the protection of human health. This is evidenced especially in the case of SO_2_, O_3_, and particulate matter when in many cases the EU legislative requirements are fulfilled but the WHO AQG are repeatedly exceeded harming human health.

## Electronic supplementary material

ESM 1 (DOCX 1735 kb)
