# Supplementary material for: Trends and patterns of air quality in Santa Cruz de Tenerife (Canary Islands) in the period 2011–2015
Source: Air Qual Atmos Health. 2017 May 17;10(8):939–54. doi: 10.1007/s11869-017-0484-x (PMC5660841; doi:10.1007/s11869-017-0484-x)
Supplement: Supplementary file 1 — (DOCX 1735 kb) [file 11869_2017_484_MOESM1_ESM.docx]

**6. Supplementary materials**

**Table S.1**. Monitoring stations, letter codes and percentages of hourly data capture per pollutant and station during the year. Green color highlights a data availability of 90% or more (or >75% for winter O_3_ measurements), valid for data quality objectives, yellow between 90% and 75% and red less than 75%. Letter codes correspond to stations in figure 1

**
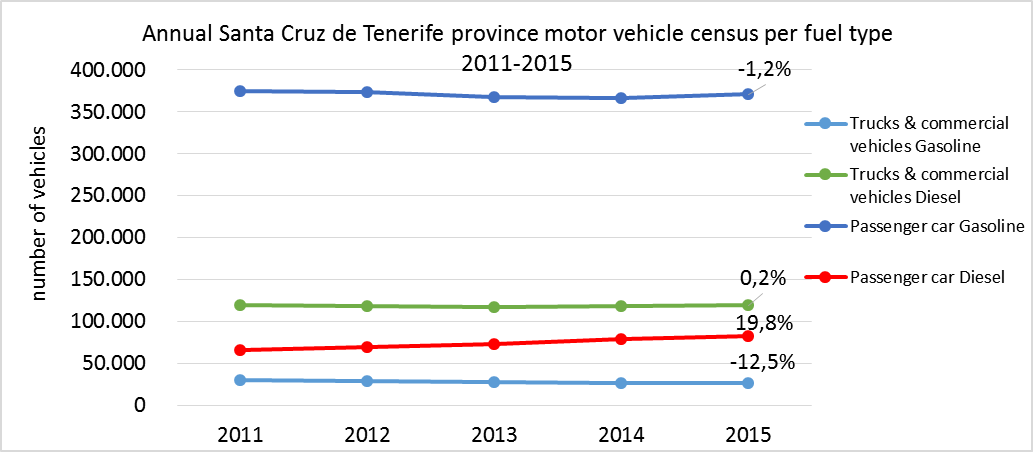
**

**Fig. S.1.** Motor vehicle census per fuel type 2011-2015 in Santa Cruz de Tenerife province. Percentages of variation in number of vehicles respect 2011 are also shown


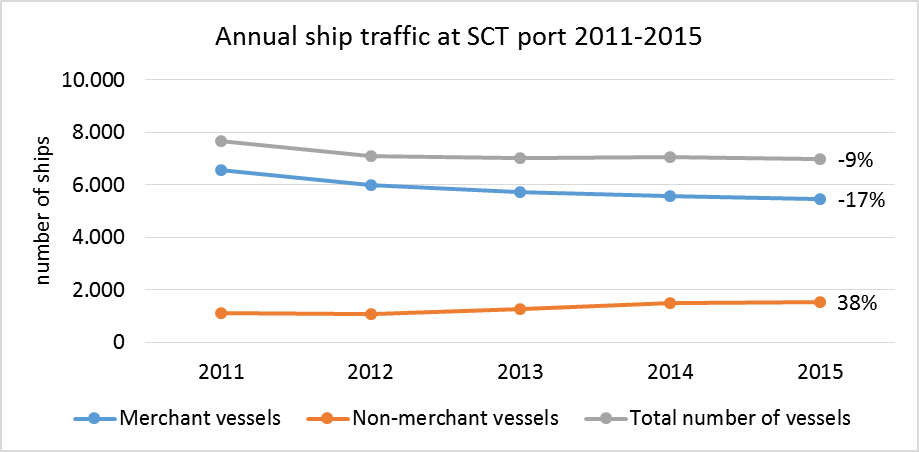


**Fig. S.2.** Ship traffic at Santa Cruz de Tenerife port. Merchant, non-merchant and total number of ships. Percentages of variation with respect to 2011 are also shown


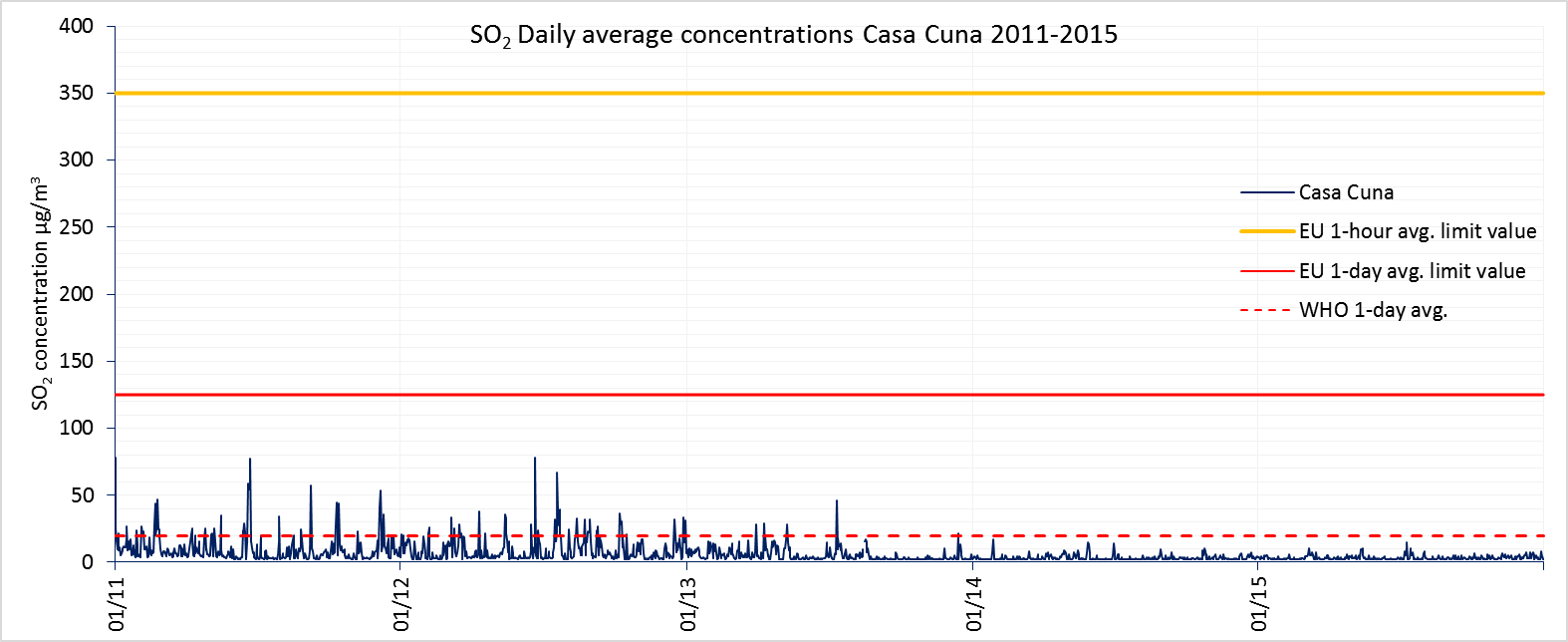


**Fig. S.3.** SO_2_ daily mean concentrations registered in Casa Cuna measuring station over 2011-2015. Hourly and daily mean limit values from the EU Directive and daily mean WHO AQG values are also shown


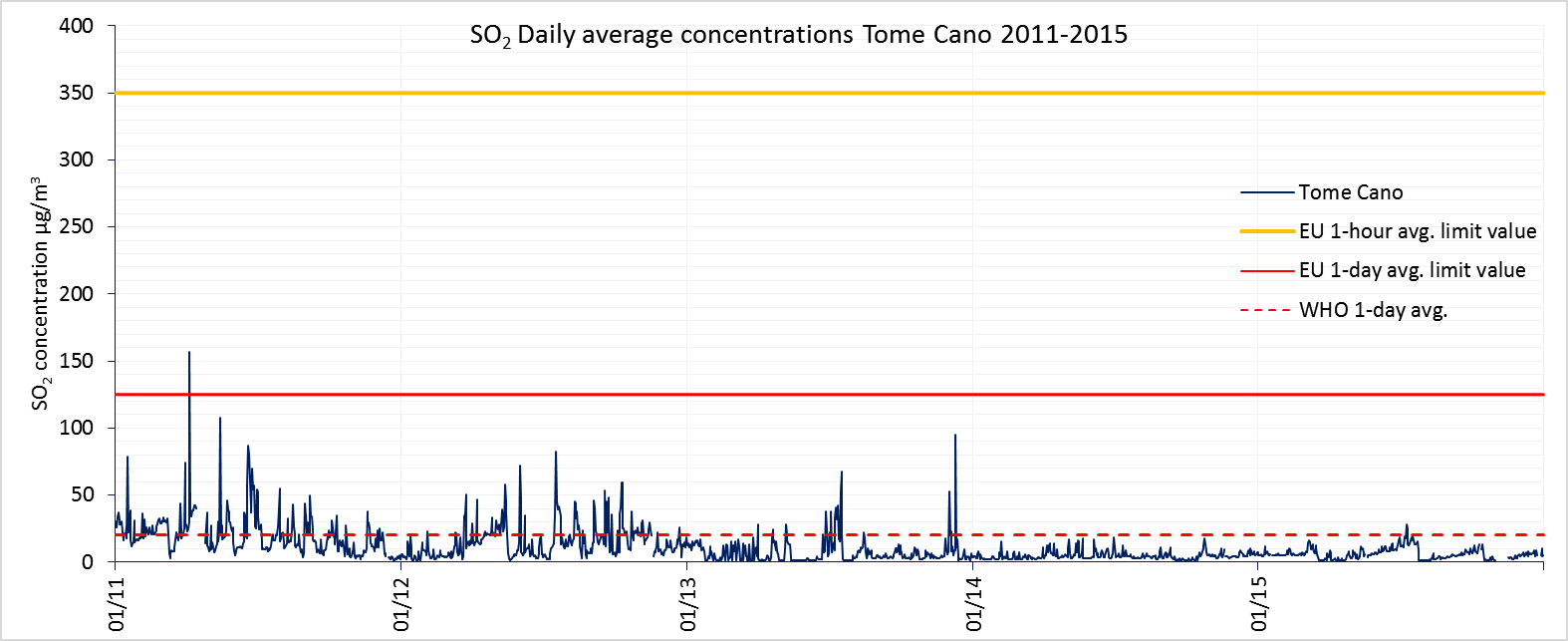


**Fig. S.4.** SO_2_ daily mean concentrations registered in Tome Cano measuring station over 2011-2015. Hourly and daily mean limit values from the EU Directive and 1-day mean WHO AQG values are also shown


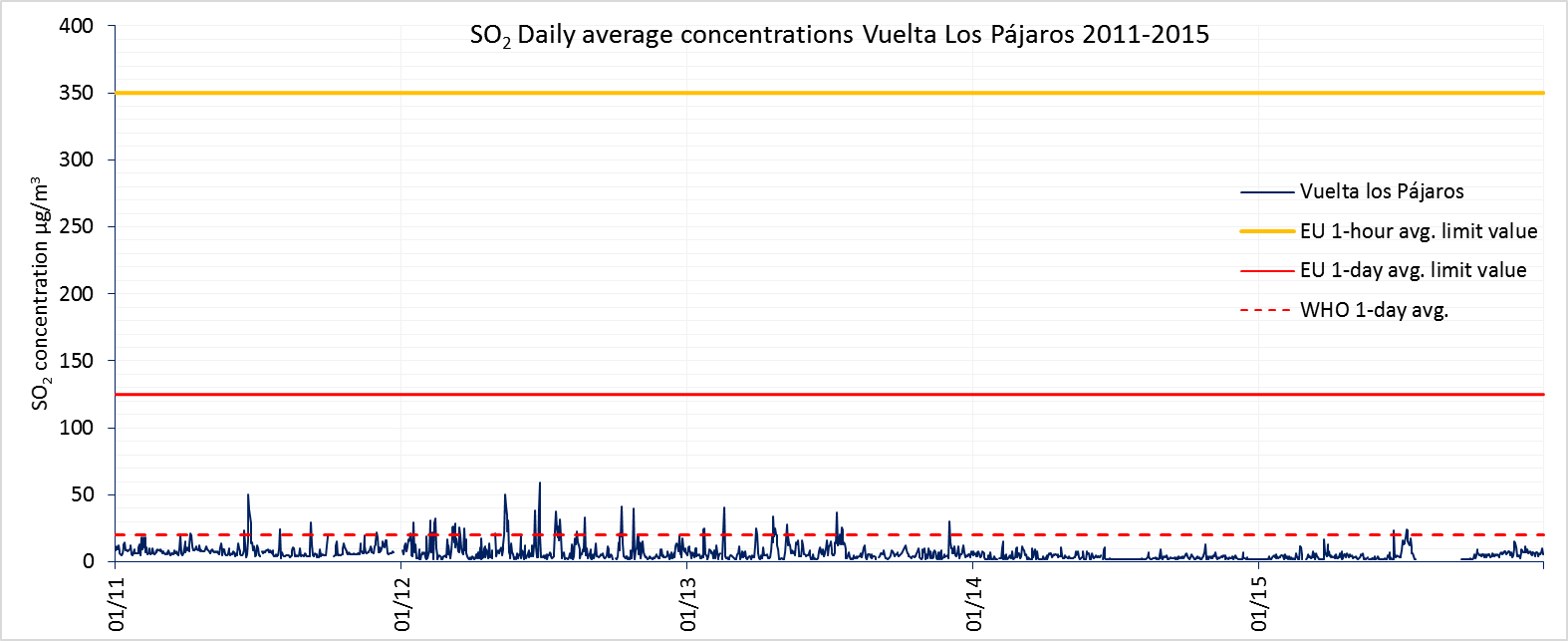


**Fig. S.5.** SO_2_ daily mean concentrations registered in Vuelta Los Pájaros measuring station over 2011-2015. Hourly and daily mean limit values from the EU Directive and 1-day mean WHO AQG values are also shown


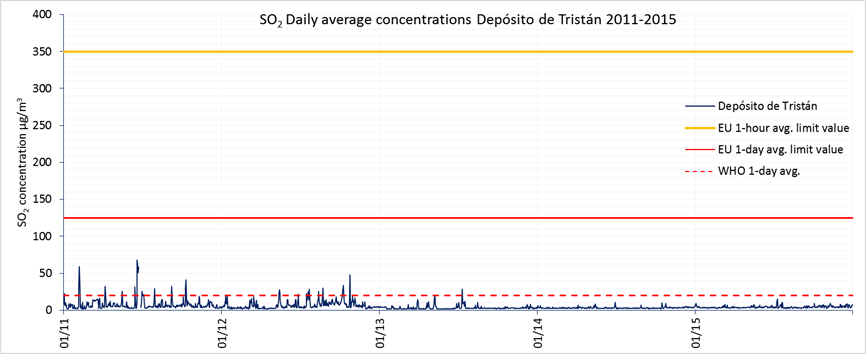


**Fig. S.6.** SO_2_ daily mean concentrations registered in Depósito de Tristán measuring station over 2011-2015. Hourly and daily mean limit values from the EU Directive and 1-day mean WHO AQG values are also shown.

**
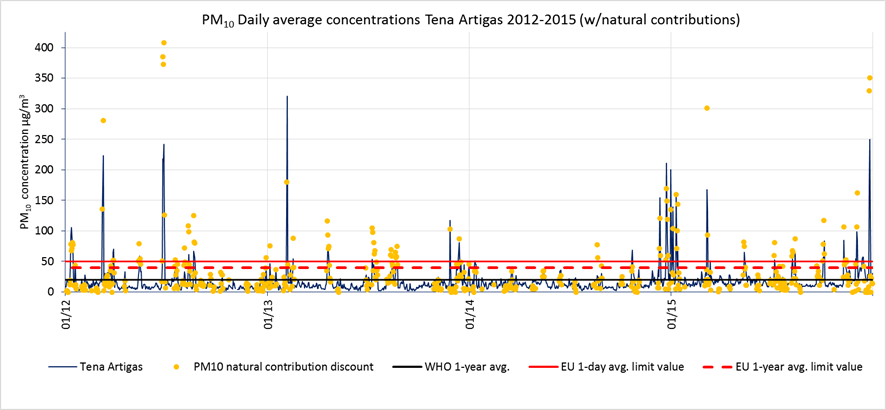
**

**Fig. S.7.** PM_10_ daily mean concentrations registered in Tena Artigas measuring station during 2012-2015. Hourly and daily mean limit values from the EU Directive and 1-year mean WHO AQG are also shown. Yellow dots are natural source contributions provided by MAPAMA 2016


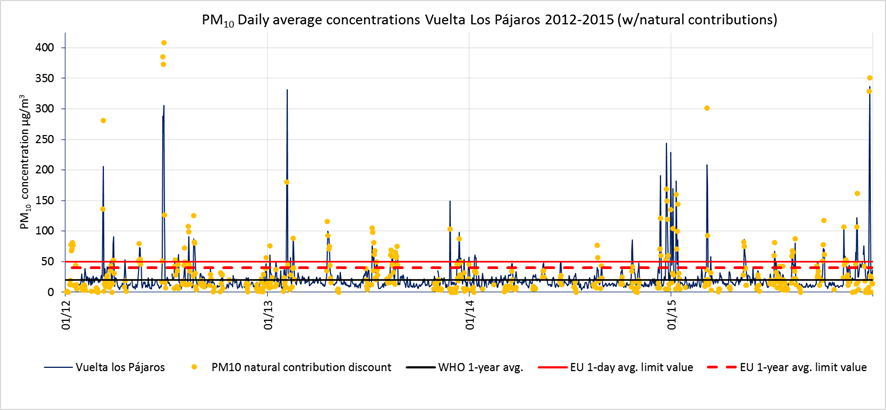


**Fig. S.8.** PM_10_ daily mean concentrations registered in Vuelta Los Pájaros measuring station during 2012-2015. Hourly and daily mean limit values from the EU Directive and 1-year mean WHO AQG are also shown. Yellow dots are natural source contributions provided by MAPAMA 2016


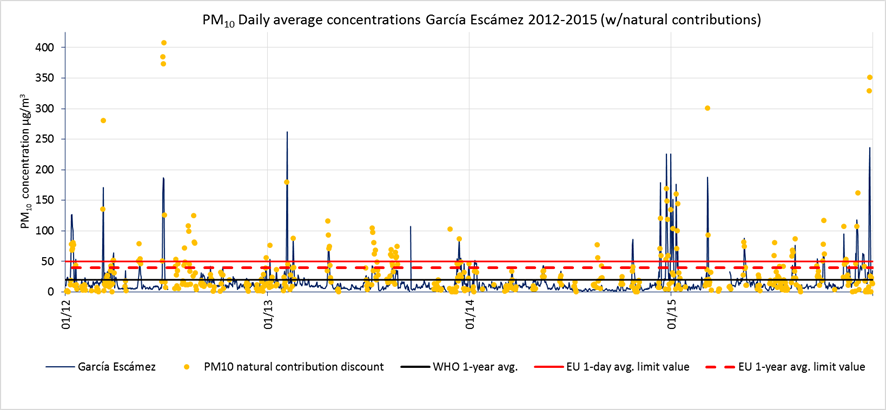


**Fig. S.9.** PM_10_ daily mean concentrations registered in García Escámez measuring station during 2012-2015. Hourly and daily mean limit values from the EU Directive and 1-year mean WHO AQG are also shown. Yellow dots are natural source contributions provided by MAPAMA 2016


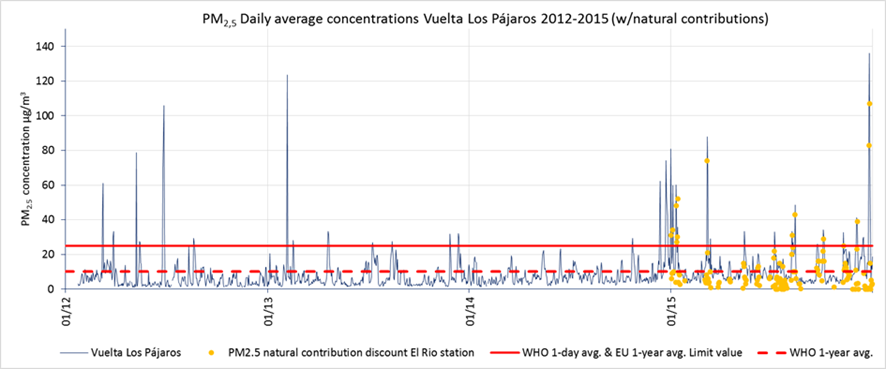


**Fig. S.10.** PM_2.5_ daily mean concentrations registered in Vuelta Los Pájaros measuring station during 2012-2015. Annual mean limit values from the EU Directive, annual and 1-day WHO AQG are also shown. From 2015 yellow dots represent the natural source contributions calculated provided by MAPAMA 2016


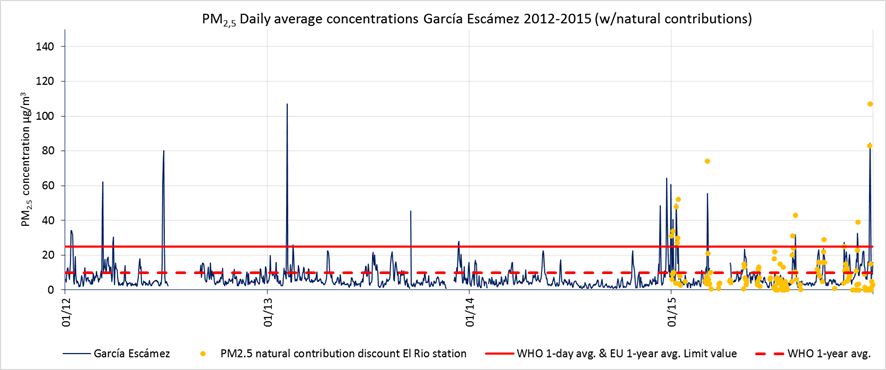


**Fig. S.11.** PM_2.5_ daily mean concentrations registered in Garcia Escámez measuring station during 2012-2015. Annual mean limit values from the EU Directive, annual and 1-day WHO AQG are also shown. From 2015 yellow dots represent the natural source contributions calculated provided by MAPAMA 2016


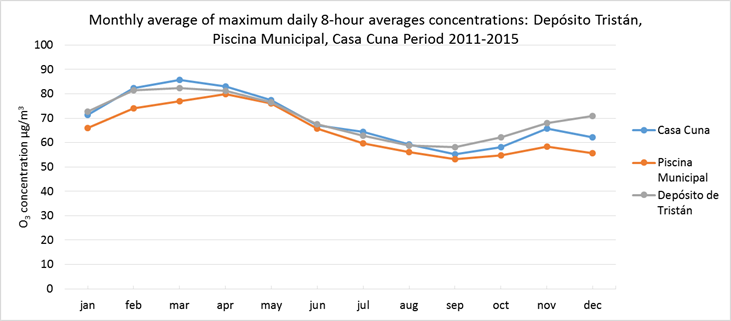


**Fig. S.12.** Monthly average of maximum daily 8-hour averages concentrations measured at Depósito Tristán, Piscina Municipal and Casa Cuna stations during 2011-2015.

| 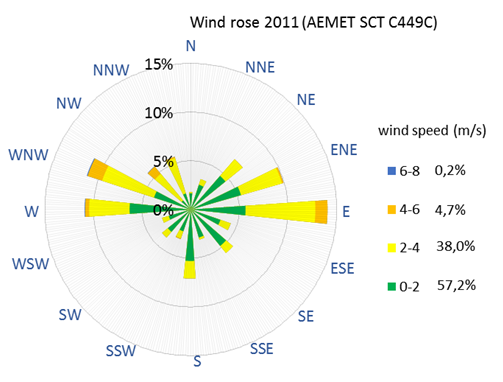 | 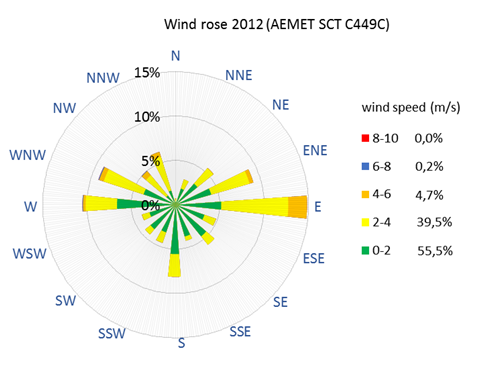 |
| --- | --- |
| 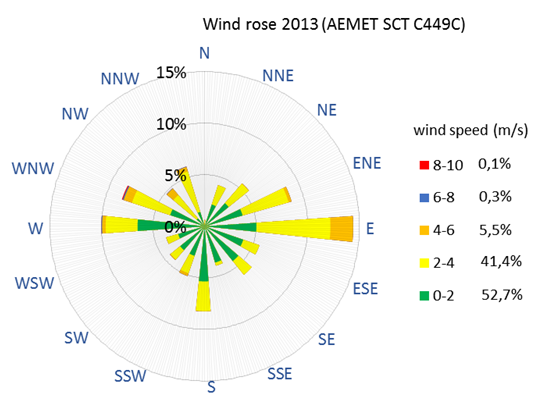 | 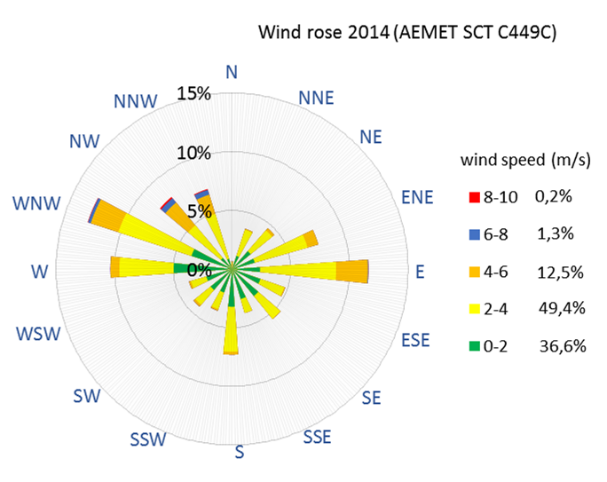 |
| 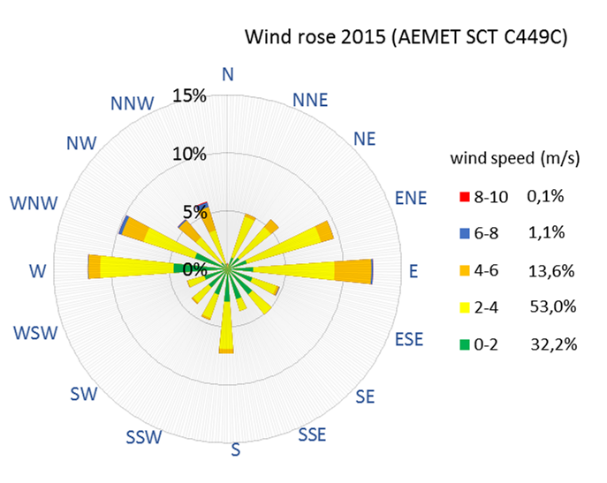 |  |

**Fig. S.13.** SCT Wind Roses during 2011-2015, measurements performed at AEMET’s weather station id. C449C. See figure 1 for city location

**Fig. S.14**. Average SO_2_ concentrations measured at Casa Cuna 2011-2015, hourly - monthly time patterns

**Fig. S.15**. Average SO_2_ concentrations measured at Piscina Municipal 2011-2015, hourly - monthly time patterns

**Fig. S.16**. Average NO_2_ concentrations measured at Casa Cuna 2011-2015, hourly - monthly time patterns

**Fig. S.17**. Average NO_2_ concentrations measured at Piscina Municipal 2011-2015, hourly - monthly time patterns
